# An organotypic slice culture model of chronic white matter injury with maturation arrest of oligodendrocyte progenitors

**DOI:** 10.1186/1750-1326-6-46

**Published:** 2011-07-05

**Authors:** Justin M Dean, Art Riddle, Jennifer Maire, Kelly D Hansen, Marnie Preston, Anthony P Barnes, Larry S Sherman, Stephen A Back

**Affiliations:** 1 Department of Pediatrics, Oregon Health & Science University, 3181 SW Sam Jackson Park Road, Portland, Oregon 97239, USA; 2 Department of Neurology, Oregon Health & Science University, 3181 SW Sam Jackson Park Road, Portland, Oregon 97239, USA; 3 Division of Neuroscience, Oregon National Primate Research Center, Oregon Health & Science University, 505 NW 185th Ave, Portland, OR 97006, USA

**Keywords:** white matter, oligodendrocyte, gliosis, astrocyte, hyaluronan, slice culture

## Abstract

**Background:**

CNS myelination disturbances commonly occur in chronic white matter lesions in neurodevelopmental and adult neurological disorders. Recent studies support that myelination failure can involve a disrupted cellular repair mechanism where oligodendrocyte (OL) progenitor cells (OPCs) proliferate in lesions with diffuse astrogliosis, but fail to fully differentiate to mature myelinating OLs. There are no *in vitro *models that reproduce these features of myelination failure.

**Results:**

Forebrain coronal slices from postnatal day (P) 0.5/1 rat pups were cultured for 1, 5, or 9 days *in vitro *(DIV). Slices rapidly exhibited diffuse astrogliosis and accumulation of the extracellular matrix glycosaminoglycan hyaluronan (HA), an inhibitor of OPC differentiation and re-myelination. At 1 DIV ~1.5% of Olig2^+ ^OLs displayed caspase-3 activation, which increased to ~11.5% by 9 DIV. At 1 DIV the density of PDGFRα^+ ^and PDGFRα^+^/Ki67^+ ^OPCs were significantly elevated compared to 0 DIV (*P *< 0.01). Despite this proliferative response, at 9 DIV ~60% of white matter OLs were late progenitors (preOLs), compared to ~7% in the postnatal day 10 rat (*P *< 0.0001), consistent with preOL maturation arrest. Addition of HA to slices significantly decreased the density of MBP^+ ^OLs at 9 DIV compared to controls (217 ± 16 *vs. *328 ± 17 cells/mm^2^, respectively; *P *= 0.0003), supporting an inhibitory role of HA in OL lineage progression in chronic lesions.

**Conclusions:**

Diffuse white matter astrogliosis and early OPC proliferation with impaired OL maturation were reproduced in this model of myelination failure. This system may be used to define mechanisms of OPC maturation arrest and myelination failure related to astrogliosis and HA accumulation.

## Introduction

Disturbances in CNS myelination are a central feature of numerous neurodevelopmental and adult neurological disorders, and are widely recognized to occur in areas of reactive astrogliosis. Although myelination disturbances frequently involve oligodendrocyte (OL) degeneration [1-3], emerging evidence supports that OL progenitor cells (OPCs) exhibit a robust regenerative response to injury. In chronic white matter lesions, OPCs proliferate but fail to fully differentiate to mature myelinating OLs, supporting the concept that failure to generate new myelin is related to arrest of oligodendrocyte maturation [4-6].

The mechanisms that mediate inhibition of OL maturation following CNS insults are largely unknown. Reactive astrogliosis is linked to OPC maturation arrest and remyelination failure in a number of conditions [7-9], and both Notch signaling and bone morphogenetic proteins induced during reactive gliosis have been implicated in these inhibitory processes [10,11]. Release of hyaluronan (HA) by reactive astrocytes also appears to be an important regulator of CNS myelination [12], and HA can arrest OPC maturation both *in vitro *and *in vivo *[13,14]. HA is a non-sulfated, protein-free glycosaminoglycan that forms an integral part of the extracellular matrix. In the CNS, HA is predominantly synthesized by astrocytes, and can accumulate in areas of chronic astrocytosis and myelination disturbance [15]. HA and its receptor CD44 are robustly expressed in white matter lesions with diffuse astrogliosis, consistent with the response observed in demyelinating lesions, traumatic spinal cord injury, vascular brain injury associated with dementia, and ischemic lesions in adult humans and rodents [14,16,17]. The molecular mechanisms by which HA inhibits OL maturation are largely unknown, and as yet, there are no well-established *in vitro *models that reproduce the major features of chronic white matter lesions.

Herein, we developed a slice culture model of reactive astrogliosis that exhibited accumulation of HA in the white matter, with associated OPC proliferation but impaired maturation. Addition of HA to this system further impaired OPC maturation, providing support for an inhibitory role of HA in OL lineage progression. This chronic white matter injury model thus provides a novel system to define mechanisms of myelination failure related to astrogliosis and disturbances in oligodendrocyte maturation.

## Results

### Organotypic slice cultures display progressive diffuse astrogliosis and HA accumulation

**Figure 1:**
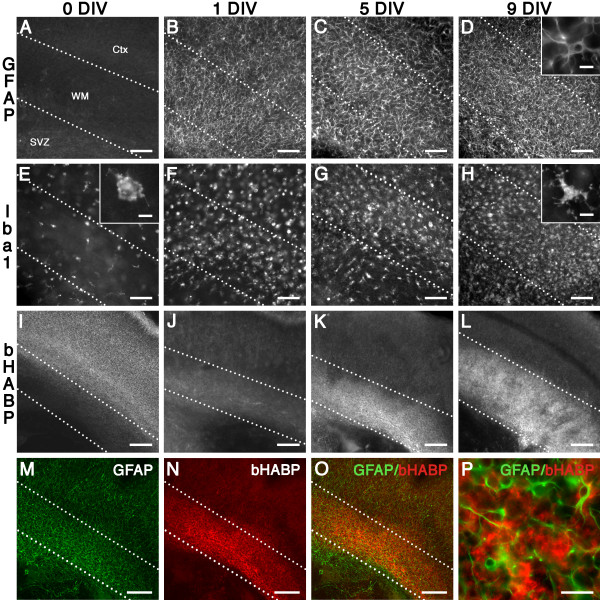
**Development of white matter gliosis in *ex vivo *slice culture model**. (A-D) Compared to 0 DIV (A), there was a marked increase in GFAP^+ ^astrocytes in the white matter at 1 DIV (B), 5 DIV (C), and 9 DIV (D), and cells exhibited a reactive phenotype (D, inset). (E-H) Compared to 0 DIV (E), there was a marked increase in Iba1^+ ^microglia/macrophages in the white matter at 1 DIV (F), 5 DIV (G), and 9 DIV (H), and cells exhibited a reactive phenotype (H, inset). (I-L) bHABP staining at 0 DIV (I) was low in the white matter in contrast to the elevated staining in the peri-neuronal nets of the cerebral cortex. Compared to 0 DIV (I), there was a progressive increase in bHABP in the white matter at 1 DIV (J), 5 DIV (K), and 9 DIV (L). (M-O) In contrast to the cortex, areas of white matter gliosis (M) were enriched in bHABP staining (N); double labeling is shown in O. (P) HA (red) exhibited a perinuclear pattern in areas of gliosis (GFAP, green). Scale: A-H, 100 μm; I-O, 200 μm; P, 25 μm; Insets D, E, H, 10 μm. Ctx, cortex; SVZ, subventricular zone; WM, white matter.

Intact whole coronal forebrain slices containing white matter and overlying cortex were cultured from postnatal day 0.5/1 (P0.5/1) rats. To investigate the glial injury response in this slice culture model, we analyzed immunohistochemical expression of GFAP (astrocytes) and Iba1 (microglia/macrophages) in the white matter at 0, 1, 5, and 9 days *in vitro *(DIV). At 0 DIV (i.e., P0.5/1 rat brain with no culture), there was negligible expression of GFAP in the white matter (Figure 1A), and low expression of Iba1 (Figure 1E) in cells that exhibited a resting morphology (Figure 1E inset). Compared to 0 DIV (Figure 1A), there was a rapid and progressive up-regulation of GFAP expression in astrocytes (Figure 1B-D) and Iba1 in microglia/macrophages (Figure 1E-H). Both cell types exhibited a reactive morphology (GFAP^+ ^cells, Figure 1D inset; Iba1^+ ^cells, Figure 1H inset). Overall, these data supported a pattern of progressive reactive gliosis.

Next, we detected HA expression with a biotinylated hyaluronan (HA) binding protein (bHABP). At 0 DIV (Figure 1I), there was low HA expression in the white matter, followed by a progressive increase from 1 DIV to 9 DIV (Figure 1J-L). Elevated HA was observed predominantly in areas of gliosis (Figure 1M-O), with a characteristic pericellular pattern of expression (Figure 1P), consistent with previous observations [14]. Hence, chronic slice cultures displayed diffuse astrogliosis and HA accumulation similar to that observed in chronic cerebral whiter matter lesions *in vivo*.

### Oligodendrocyte (OL) survival and progenitor responses in white matter of organotypic slice cultures

**Figure 2:**
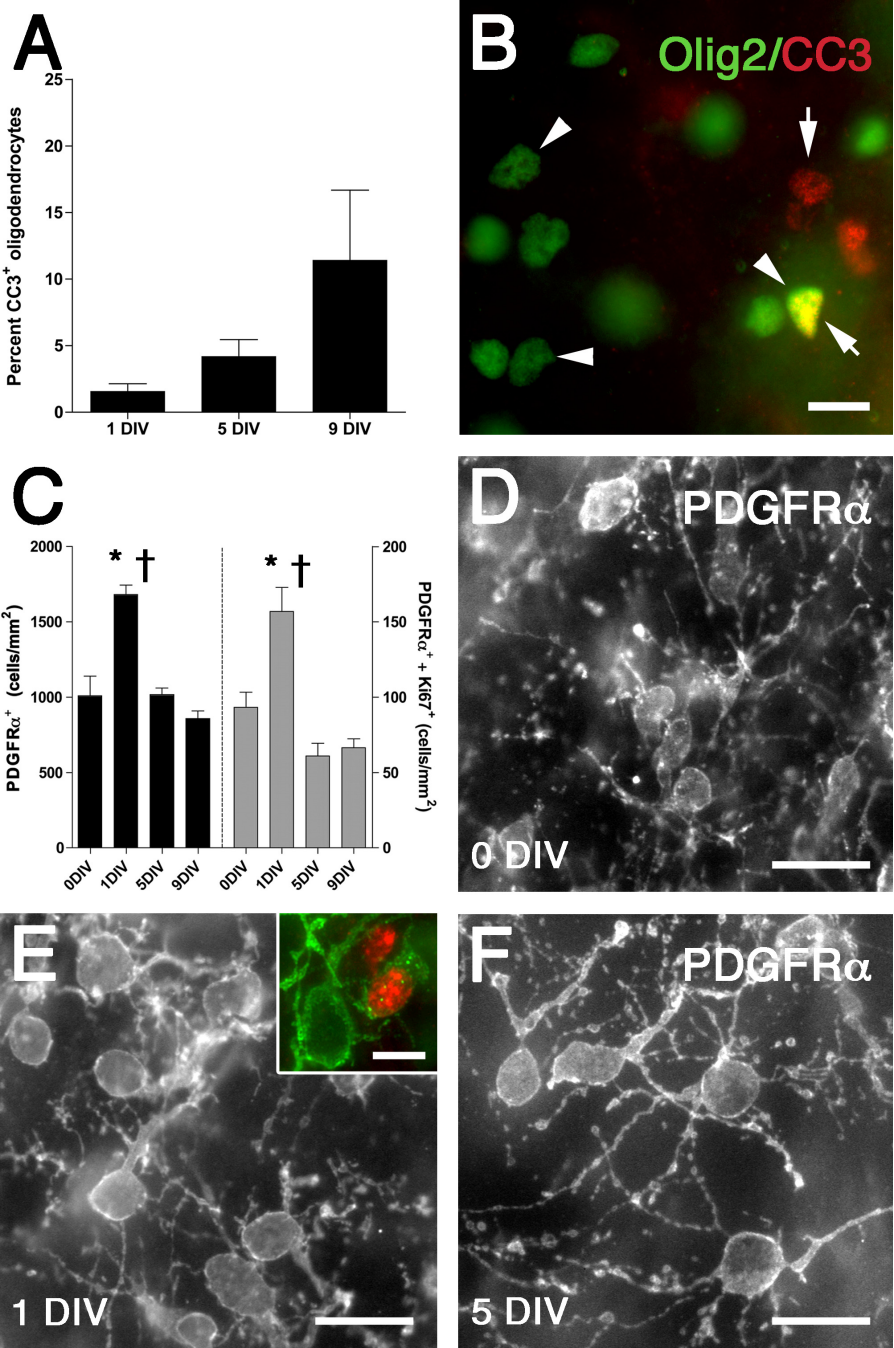
**Oligodendrocyte (OL) survival and oligodendrocyte progenitor proliferation in the white matter of *ex vivo *slice culture model**. (A) Percentage of total OL lineage cells in the white matter that labeled with cleaved caspase-3^+ ^(CC3). Data are mean ± SEM (n = 3-4 slices per time point). (B) Example of CC3^+ ^(red, arrows) and Olig2^+ ^(green, arrowheads) cells (co-localized cells are shown in yellow). (C) Density of PDGFRα^+ ^(black bars, left axis) and co-localized PDGFRα^+^/Ki67^+ ^OPCs in the white matter. Data are mean ± SEM (n = 6 slices per time point from two independent experiments). *P < 0.01 (ANOVA followed by Tukey's multiple comparison test; uncorrected data). †*P *< 0.01 (ANCOVA followed by Tukey's multiple comparison test; data corrected for atrophy). (D-F) PDGFRα^+ ^OPCs in the white matter at 0 DIV (D), 1 DIV (E) and 5 DIV (F). The inset in E shows a PDGFRα^+ ^(green)/Ki67^+ ^(red) double labeled OPC. Scale: B, D-F, 20 μm; E inset, 10 μm.

To investigate survival of the total OL lineage in this slice culture model, we examined the density of cleaved caspase-3 (CC3)-positive cells (Olig2^+^) in the white matter at 1 DIV, 5 DIV, and 9 DIV (Figure 2A, B). Approximately 1.5% of all Olig2^+ ^cells were positive for CC3 at 1 DIV. There was a progressive but non-significant increase to ~11.5% by 9 DIV.

To examine the response of OPCs in the white matter of this slice culture model, we examined the density of PDGFRα^+ ^OPCs and their co-localization with the proliferation marker Ki67 at 0 DIV, 1 DIV, 5 DIV and 9 DIV (Figure 2C). At 1 DIV, there was a significant increase in the density of both PDGFRα^+ ^cells and PDGFRα^+^/Ki67^+ ^double-labeled cells compared to 0 DIV (ANCOVA + Tukey's test; *P *< 0.01, for both), consistent with OPC proliferation. By 5 DIV and 9 DIV the density of both PDGFRα^+ ^cells and PDGFRα^+^/Ki67^+ ^double-labeled cells returned to 0 DIV levels. With increasing time in culture, PDGFRα^+ ^OPCs exhibited a reactive-type of morphology, with increased cell body size and process thickness, as well as more extensive process arborization and complexity (Figure 2D-F). Hence, this slice culture model was associated with an acute phase of rapid OPC proliferation resulting in a net expansion in the OPC pool, followed by a delayed phase of degeneration.

### Delayed OPC maturation in white matter of organotypic slice cultures

**Figure 3:**
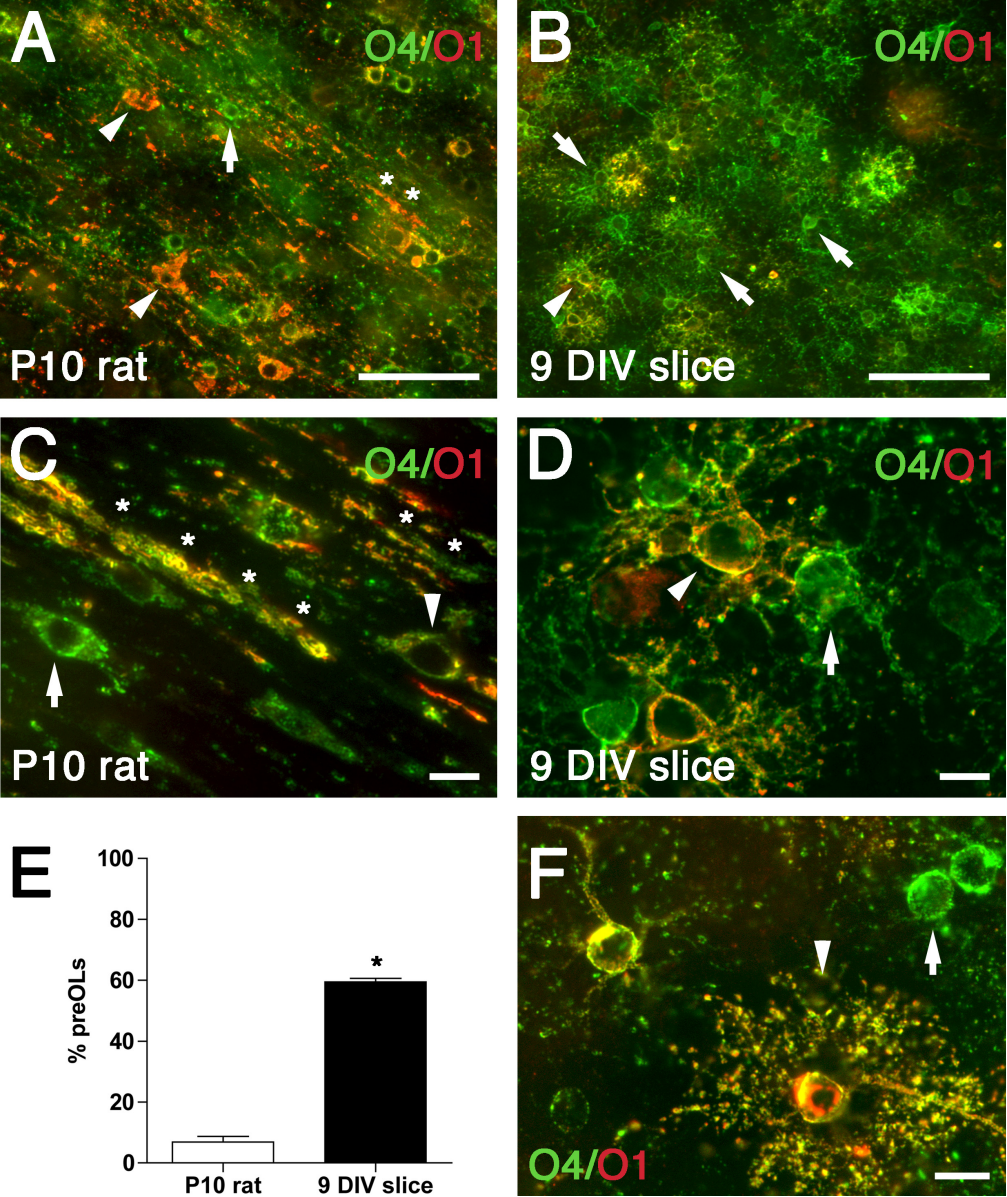
**Oligodendrocyte maturation is delayed in the white matter of the *ex vivo *slice culture model compared to normal white matter development *in vivo***. Representative photomicrographs of preOLs (green; arrows) and immature OLs (yellow; arrowheads) double-labeled with O4 (green) and O1 (red) antibodies in the P10 rat (A, C) and the white matter of the 9 DIV slice (B, D, F). (A, C) The P10 rat white matter contained numerous immature OLs (arrowheads) and myelin sheaths (asterisk). (B, D) Slice cultures contained predominantly preOLs (green; arrows) with occasional immature OLs (yellow; arrowheads). Both preOLs and immature OLs in the slice cultures showed a reactive morphology, with extensive process extension and cytoplasmic swelling when compared with normal brain at P10 (C). (E) Percentage of preOLs in the white matter of P10 rat (white bar) *vs. *organotypic slice culture at 9 DIV (Black bar)*. *Data are mean ± SEM (9 DIV slice data: n = 11 slices from three independent experiments; P10 rat data: n = 6 animals). *P < 0.0001 (unpaired two-way t-test). (F) Some O4^+^/O1^+ ^OLs in slice culture showed a highly branched morphology (arrowhead) suggestive of a mature OL; arrow indicates a preOL. Scale: A, B, 50 μm; C, D, F, 10 μm.

To determine the timing of OPC maturation, we quantified the relative percentages of late oligodendrocyte precursors (preOLs; O4^+^/O1^-^) and immature oligodendrocytes (immature OLs; O4^+^/O1^+^) in slice cultures at 9 DIV compared to the normal rat brain at an equivalent postnatal age (P10). Rat brains at P10 only expressed 7 ± 2% preOLs in the white matter (Figure 3A, E). Most O4^+^/O1^+ ^immature OLs displayed a reduced process arbor and extensive early myelination (Figure 3C). By contrast, there were 60 ± 1% preOLs in the white matter in this slice culture model at 9 DIV (Figure 3B, E; *P *< 0.0001). Interestingly, both preOLs and immature OLs displayed a reactive morphology, with a hypertrophic cell body and an extensive arbor of processes (Figure 3D) relative to normal brain at P10 (Figure 3C). Some O4^+^/O1^+ ^OLs showed a highly branched morphology consistent with mature OLs (Figure 3F). Hence, the maturation of OPCs in this slice model was markedly delayed relative to the normal rat brain, with arrested OL maturation at the preOL stage.

### Timing of myelin onset in white matter of organotypic slice cultures

**Figure 4:**
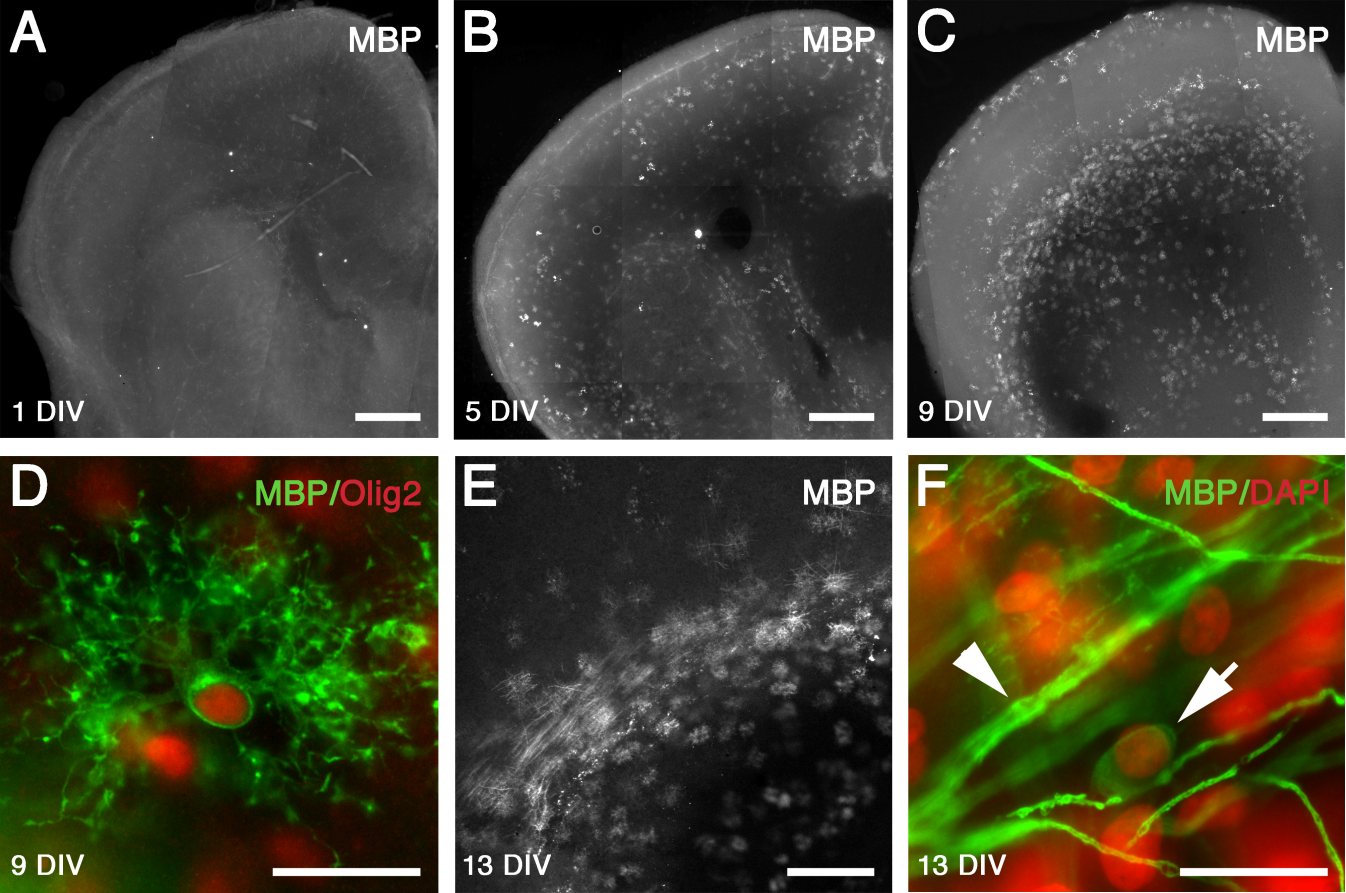
**Timing of myelin onset in white matter of *ex vivo *slice culture model**. MBP expression at 1 DIV (A), 5 DIV (B), and 9 DIV (C) in white matter of organotypic slice cultures. There was a progressive increase in MBP expression in the white matter with time in culture. (D) At 9 DIV, robust expression of individual MBP positive cells was observed, which were co-localized with the oligodendrocyte nuclear transcription factor Olig2 (MBP: green, Olig2: red). (E) Evidence of onset of myelination in the white matter at 13 DIV. (F) Oligodendrocyte (arrow; DAPI: shown as pseudo-color in red) showing wrapping of axons stained with MBP (green; arrowhead). Scale: A-C, 500 μm; D, F, 20 μm; E, 250 μm.

We examined the immunohistochemical expression of MBP in the white matter at 1 DIV, 5 DIV, 9 DIV, and 13 DIV. At 1 DIV there were no MBP^+ ^cells in the slice cultures (Figure 4A). By 5 DIV a low number of MBP^+ ^mature OLs were observed (Figure 4B). By 9 DIV there was a marked increase in expression of individual MBP^+ ^cells (Figure 4C). The MBP^+ ^cells at 9 DIV were highly branched but largely occupied individual domains, and co-localized with the nuclear oligodendrocyte marker Olig2 (Figure 4D). At 13 DIV a small number of MBP^+ ^cells appeared to be initiating myelination (Figure 4E, F). Despite the presence of a subpopulation of mature OLs in the white matter, there was thus a pronounced reduction in myelination compared to the normal rat brain at P10 (Figure 3C) or P14 [18,19].

### Exogenous hyaluronan impairs oligodendrocyte maturation in white matter of organotypic slice cultures

**Figure 5:**
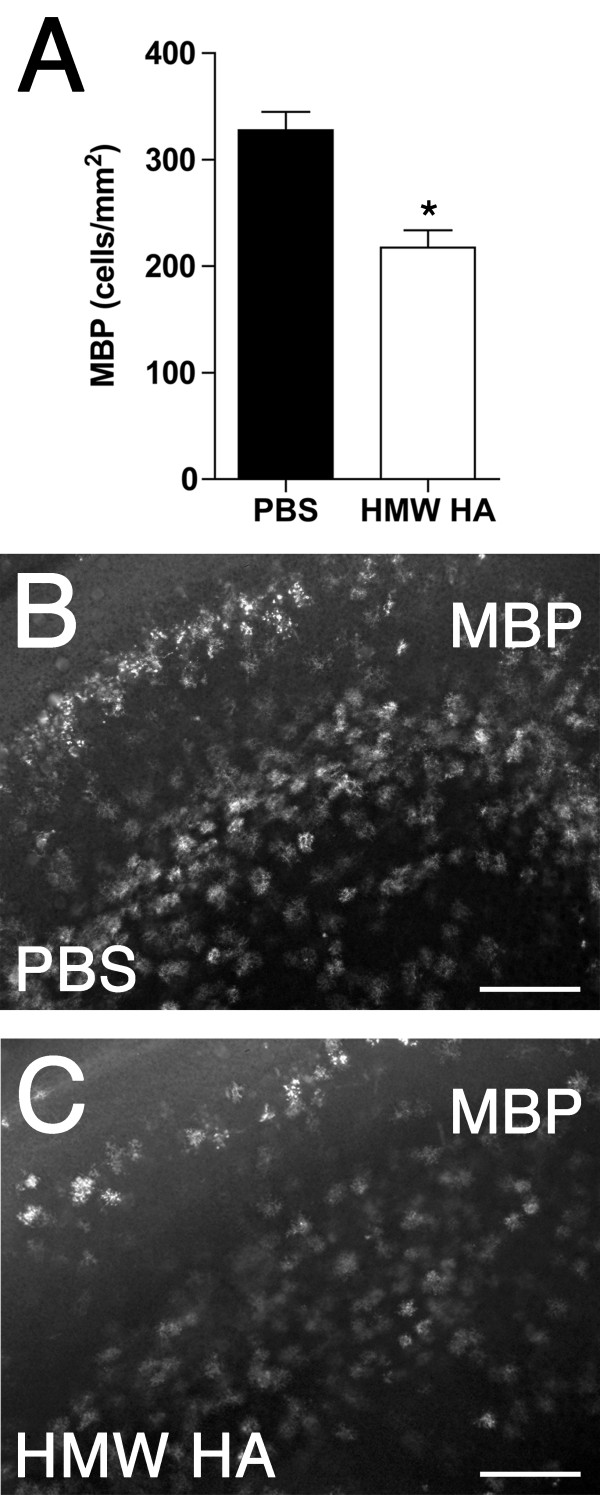
**Hyaluronan impairs oligodendrocyte maturation in organotypic slice cultures**. (A) Density of MBP^+ ^oligodendrocytes in the white matter at 9 DIV in PBS (Black bar) and HA (white bar) treated slices. Data are mean ± SEM (n = 9 slices per time group from three independent experiments). *P < 0.0005 (unpaired two-way t-test). (B, C) Representative photomicrographs of 9 DIV slices stained with MBP in the PBS (B) and HA (C) treatment groups. Scale: B, C, 100 μm.

We added high molecular weight HA to this slice culture system to examine its role in regulating oligodendrocyte maturation. At 9 DIV there was a significant decrease in the density of MBP^+ ^oligodendrocytes in the white matter in slices treated with HA compared to those treated with PBS (~34%; 217 ± 16 *vs. *328 ± 17 cells/mm^2^, respectively; *P *= 0.0003; Figure 5A-C). Hence, exogenous HA further reduced the level of OL maturation relative to that in untreated chronic slices where OL maturation was already markedly lower than equivalent age uninjured control animals.

## Discussion

The normal regulation of oligodendrocyte (OL) maturation and myelination in the CNS is critical for normal vertebrate function, as well as to promote recovery following white matter injury. Dissociated OL cultures have provided important information on many aspects of these processes. Nevertheless, there are no well-established *in vitro *models that accurately model the chronic gliotic lesions often observed in disorders with myelination failure. There is now increasing interest in the use of organotypic slice cultures for studies of OL biology and myelination due to retention of multi-cellular interactions and the ease of manipulation [20-22]. However, we found that organotypic slice cultures display features consistent with a chronic white matter injury *in vivo*, which included white matter gliosis, cell death, OPC proliferative and reactive responses and arrested OL lineage maturation. Hence, when employing a chronic slice culture model for studies of normal oligodendroglial biology or myelination, these chronic injury responses should be considered.

Similar to recent *in vivo *studies examining the chronic OL response to white matter injury, we found rapid and progressive reactive astrocytosis and microglia/macrophage accumulation in the white matter of slice cultures, as well as an early proliferative response of OPCs. This expanded pool of OPCs exhibited a reactive morphology, delayed OL degeneration, and impaired maturation of preOLs to OLs. Delayed OPC maturation did not appear to be region-specific and cortical OPC maturation by 9 DIV also was delayed relative to the extensive myelination that occurs in vivo by postnatal day 10. These *in vitro *responses are very similar to those first described in neonatal rodents following hypoxia-ischemia, where despite extensive OL degeneration, there was a rapid proliferative response of OPCs, subsequent failure of preOLs to mature in chronic astrogliotic lesions, and persistent myelination deficits [4]. A role for OL maturation arrest in developmental myelination disturbance is supported by more recent studies in preterm fetal sheep following hypoxia-ischemia [23] and in preterm human autopsy cases with chronic white matter injury (Buser et al., submitted). OPCs also accumulate but fail to mature to myelinating OLs in chronic demyelinated lesions in multiple sclerosis patients [24-26]. In a recent postmortem study of brains from human cases of age-related cognitive decline associated with vascular brain injury, a significant increase in the total pool of OLs was correlated with changes in MRI-defined diffusion characteristics consistent with white matter myelin deficits [27].

Our slice culture system exhibited a progressive increase in expression of HA in the white matter with time in culture, while addition of exogenous HA further inhibited OL maturation. These data suggest a role for endogenously released HA in mediating the impairment of OL maturation observed in this system, and support previous studies where addition of HA reversibly impaired maturation of cultured OPCs and inhibited remyelination after lysolethicin-induced white matter demyelination [13,14]. Accumulation of HA was also reported following traumatic spinal cord injury [16] and middle cerebral artery occlusion [28] in adult rats, and in stroke-affected brain regions in adult humans [29], although myelination deficits were not examined. The increase in white matter HA in the slice cultures was likely a response to the reactive astrogliosis, because in the CNS, HA is predominantly produced by astrocytes [30]; however, we cannot discount a role of other CNS glia. The reactive gliosis observed in this system was likely a response to neuronal and glial degeneration secondary to generation of the tissue slices.

## Conclusions

This model provides a novel system to define mechanisms that regulate disturbances in oligodendrocyte maturation and myelination failure related to chronic CNS astrogliosis. Future studies will utilize this model to determine the signaling pathways by which HA regulates oligodendrocyte development in the setting of chronic white matter injury.

## Methods

### Postnatal brain slice preparation and culture

All animal procedures were approved by the OHSU Institutional Animal Care and Use Committee (IACUC) according to the NIH Guide for the Care and Use of Laboratory Animals. Timed pregnant Sprague-Dawley (SD) rats were purchased from Charles River (Hollister, CA, USA). Whole forebrain coronal slices (300 μm; collected at the level of the rostral corpus callosum and anterior septal nuclei; 3 adjacent slices from each brain) collected from postnatal day (P) 0.5/1 rat pups were used to prepare organotypic cultures according to a previous method [31], with modifications. Brains were embedded in 1.5% low melting point agar (Fischer Scientific, Fair Lawn, NJ, USA) and sectioned into sterile ice-cold complete Hank's balanced salt solution (HBSS, Ca^2+^/Mg^2+ ^free [Invitrogen Co., Carlsbad, USA], supplemented with D-glucose [30 mM), HEPES buffer [2.5 mM], CaCl_2 _[1 mM], MgSO_4 _[1 mM], NaHCO_3 _[4 mM], and 0.001% phenol red [Sigma-Aldrich Co., St. Louis, MO, USA]) using a VTS 1600 vibrating microtome (Leica Microsystems Inc., Buffalo Grove, USA). Isolated slices were transferred onto 0.4 μm porous membrane cell culture inserts (Becton Dickinson, Franklin Lakes, NJ, USA) that were pre-coated with laminin/poly-D-lysine (Sigma-Aldrich Co.), and cultured in slice culture media (Basal Medium Eagle [Invitrogen Co.], supplemented with complete HBSS [25% v/v], D-glucose [27 mM], penicillin [100 U/mL], streptomycin [100 U/mL], glutamine [1 mM; Sigma-Aldrich Co.], and 5% horse serum [New Zealand origin, heat inactivated; Invitrogen Co.]). Slices were incubated at 37°C/5% CO_2_, and the growth medium changed daily. In pilot experiments we determined that 5% serum resulted in optimal acute survival of the slices when compared to 25% serum, as previously reported [21].

### Time course experiments

Slices were collected at 1, 5, 9, or 13 days *in vitro *(DIV), fixed in 4% paraformaldehyde (PFA; 0.1 M phosphate buffered saline [PBS]) for 1 h at RT, and washed thoroughly in PBS prior to immunohistochemical staining. As controls, slices were fixed immediately after cutting (0 DIV; i.e., no culture).

### Hyaluronan experiments

High molecular weight hyaluronan (1.59 × 10^6 ^Da; Seikagaku Co., Tokyo, Japan) was dissolved in sterile PBS (5 mg/mL), and then added daily to fresh slice growth medium (final concentration, 100 μg/mL) from 0 DIV until 9 DIV [14]. Slices were then processed for immunohistochemistry as described.

### Immunohistochemistry

Single- or double-labeling immunofluorescence was performed on sections in 24-well plates. Antibodies and dilutions are shown in Table 1. For PDGFRα/Ki67 double labeling and O4 and O1 antibody double labeling, sections were blocked for 1 h in PBS containing 5% normal goat serum (NGS), and then with primary antibodies diluted in PBS/3% NGS for 72 h at 4°C [4]. For all other antibodies, sections were incubated in 0.01 M citrate buffer (pH 6.0) at 85°C for 20 min, left to cool for 20 min, and then washed three times in PBS. Blocking and primary antibody incubation was performed as above, with the addition of 0.4% triton X-100 (Sigma-Aldrich Co.). For secondary detection, all sections were washed three times in PBS, and incubated with appropriate AlexaFluor fluorescent dye conjugated secondary antibodies (1:500, all raised in goat; Invitrogen Co.). Nuclei were counterstained with DAPI (Invitrogen Co.). No-primary-control studies for all antibodies exhibited no positive staining. Sections were mounted with Vectashield fluorescent mounting medium (Vector Laboratories, Inc., Burlingame, CA, USA).

**Table 1:** Antibodies and markers

Antigen	Host	Marker	Dilution	Supplier
Biotinylated hyaluronan binding protein (bHABP)	Bovine	Hyaluronan	1:200	Associates of Cape Cod, Inc., East Falmouth, MA

Cleaved caspase-3 (CC3)	Polyclonal Rabbit	Apoptotic cells	1:500	Cell Signaling Technology, Danvers, MA

GFAP	Polyclonal Rabbit	Astrocytes	1:500	Dako North America, Inc., Carpinteria, CA

Iba1	Polyclonal rabbit	Microglia/macrophage	1:500	Wako Chemicals USA Inc., Richmond, VA

Ki67	Monoclonal mouse	Cell Cycle Activation	1:200	Novocastra, Buffalo Grove, IL

MBP	Mouse	Mature OL	1:500	Covance, Princeton, NY

O_1_	Monoclonal mouse IgM	Immature/mature OL	1:3000	Dr. Rashmi Bansal (University of Connecticut Health Center, Farmington, CT)

Biotinylated O_4_	Monoclonal mouse IgM	Late OL progenitor/immature OL	1:500	Research Genetics, Huntsville, AL

PDGFRα	Polyclonal rabbit	OL progenitor	1:1000	R&D Systems, Minneapolis, MN

Olig2	Mouse	Oligodendrocyte (OL)	1:250	Dr. John Alberta (Dana-Farber Cancer Institute, Boston, MA

### Cell quantification

The white matter of cultured slices was analyzed using a Leica DMIRE2 inverted fluorescence microscope (Leica Microsystems Inc., Buffalo Grove, IL, USA) coupled to a Stereoinvestigator stereology system (MBF Bioscience, Williston, VT, USA). For each slice, the entire white matter (defined by DAPI-staining) region of interest (ROI) was traced at 5 × magnification. Using the software to maintain white matter boundaries, cell counts were performed using the optical fractionator probe (Grid size, 300 × 400 μm; Counting frame, 30 × 30 μm; z-depth 20 μm) at 40 × magnification in a minimum of 10 randomly selected white matter fields per slice. The slice thickness was also measured at each counting site. Cell density (mm^2^) was calculated by the formula: [cell counts/(number of fields × counting frame area (mm^2^))].

### Statistics

An unpaired two-tailed t-test was used to compare percentage OLs between normal rat brain and the slice cultures. One-way analysis of variance (ANOVA) followed by Tukey's multiple comparison test was used to assess changes in percentage CC3^+ ^OLs in the white matter over time in culture. To determine the effect of slice atrophy on PDGFRα cell density measurements, we quantified mean white matter volume. For each slice, white matter volume was calculated from white matter ROI area × mean white matter thickness (determined from all count sites) as acquired during cell counting. One-way analysis of variance (ANOVA) followed by Tukey's multiple comparison test was first run to determine relative atrophy with time in culture. There was a significant overall effect of group (*P *< 0.01), with a significant decrease in white matter volume at 5 DIV and 9 DIV compared to other ages (white matter volume, mm^3^: 0 DIV, 0.26 mm^3^; 1 DIV, 0.29 mm^3^; 5 DIV, 0.18 mm^3^; 9 DIV, 0.18 mm^3^; *P *< 0.05), which was attributed to a shrinkage of the white matter ROI area rather than in the z-plane of the slice (data not shown). Because of this reduction in white matter volume, we accounted for degree of atrophy by using white matter volume as a covariate when comparing cell density measurements between time in culture using a one-way analysis of covariance (ANCOVA) followed by Tukey's multiple comparison test. Cell density data were presented uncorrected for atrophy. A *P*-value less than 0.05 was considered statistically significant. All data are presented as mean ± standard error of the mean (SEM), with significance indicated for both ANOVA and ANCOVA analyses (For figure 2, *P < 0.01 refers to analysis of uncorrected data by ANOVA; †*P *< 0.01 refers to analysis of data corrected for slice atrophy by ANCOVA).

## Competing interests

The authors declare that they have no competing interests.

## Authors' contributions

JD designed the study, performed slice culture experiments, data collection, and statistical analyses, and drafted the manuscript with SB. AR and AB assisted in study design and model development. JM and KH performed slice culture immunohistochemistry and data collection. MP prepared HA and verified its quality, and assisted with HA labeling protocols. LS and SB conceived of the study, and participated in its design and coordination. All authors read and approved the final manuscript.
